# A New Quantum Blind Signature Scheme with BB84-State

**DOI:** 10.3390/e21040336

**Published:** 2019-03-28

**Authors:** Feng-Lin Chen, Zhi-Hua Wang, Yong-Mo Hu

**Affiliations:** 1 School of Mathematics and Computation Science, Anqing Normal University, Anqing 246133, China; 2 School of Mathematical Sciences, University of Science and Technology of China, Hefei 230026, China

**Keywords:** BB84-state, quantum fingerprint, quantum encryption algorithm, quantum blind signature, unconditional security

## Abstract

The blind signature is widely used in cryptography applications because it can prevent the signer from gaining the original message. Owing to the unconditional security, the quantum blind signature is more advantageous than the classical one. In this paper, we propose a new provable secure quantum blind signature scheme with the nonorthogonal single-photon BB84-state and provide a new method to encode classical messages into quantum signature states. The message owner injects a randomizing factor into the original message and then strips the blind factor from the quantum blind signature signed by the blind signer. The verifier can validate the quantum signature and announce it publicly. At last, the analytical results show that the proposed scheme satisfies all of the security requirements of the blind signature: blindness, unforgeability, non-repudiation, unlinkability, and traceability. Due to there being no use of quantum entanglement states, the total feasibility and practicability of the scheme are obviously better than the previous ones.

## 1. Introduction

The security of classical signature cryptography depends on solving some difficult mathematical problems, such as factoring large integers and solving the discrete logarithm. It is known that these problems will become rather simple with the emergence of quantum computers. The quantum algorithm proposed by Shor [1] in 1994 can solve the problem of integer factorization in polynomial time. Accordingly, quantum cryptography will make a revolutionary impact on the classical one. One of the known examples of quantum cryptography is the quantum key distribution (QKD) [2,3,4,5], which offers a solution of the shared key exchange with information-theoretical security. Quite a few branches of QKD have attracted a great deal of attention, and many effective results have been proposed, including quantum private query (QPQ) [6,7,8], quantum digital signature (QDS) [9,10,11,12,13] and so on.

The first QDS scheme, which is analogous to the classical Lamport’s signature scheme, was proposed by Gottesman et al. [9] in 2001. In 2002, Zeng et al. [10] first proposed the arbitrated QDS scheme with GHZstates based on symmetric cryptography. In 2014, Dunjko et al. [11] proposed the first QDS scheme with no quantum memory, which made the quantum signature feasible and practicable under the current quantum technology. Wallden et al. [12] presented a QDS scheme with quantum-key-distribution components in 2015. In 2016, Amiri et al. [13] proposed a QDS scheme that did not require trusted quantum channels and only relied on secret shared keys generated using QKD. With the proposal of the measurement device-independent (MDI) QKD by Lo et al. [14], Puthoor et al. [15] first presented an MDI-QDS scheme, which is secure against all detector side-channel attacks. In 2017, Yin et al. [16] and Roberts et al. [17] made the attempt to implement experimentally the MDI-QDS.

The blind signature was first proposed by Chaum [18] in 1982. The blind signature can effectively prevent the blind signer from getting the original message because of its blindness, so it has a wide range of applications in the fields of E-commerce and block-chain. So far, some quantum blind signature (QBS) schemes [19,20,21,22,23,24,25,26,27,28,29,30,31,32,33] have been presented. In 2009, Wen et al. [19] first proposed the weak QBS scheme based on EPRpairs. In 2010, Su et al. [20] proposed a QBS scheme based on EPR with two-state vector formalism, and then, Yang et al. [21] pointed out some attacks on Su’s scheme [20] and proposed an enhanced one. However, Zhang et al. [22] declared that the dishonest signer could obtain some secret keys in Yang’s improved scheme [21]. In 2014, Khodambashi et al. [23] proposed a sessional QBS based on EPR, where the message signature cannot be forged by the dishonest verifier. In 2015, Shi et al. [24] proposed a new QBS scheme with unlinkability based on EPR and quantum teleportation. In 2017, Luo et al. [25] pointed out a security loophole of forgery in Shi’s QBS scheme [24]. With the χ-type entangled states, Yin et al. [26] proposed a QBS scheme in 2012. With the GHZ states, Wang et al. [27] proposed a QBS scheme in 2013. Zuo et al. [28] found that the dishonest verifier could forge the blind signature in [19,26,27]. Accordingly, Zuo et al. [28] and Ribeiro et al. [29] advised that a trusted center should be involved in QBS schemes. Based on offline trusted repositories, Ribeiro et al [29] presented a perfectly secure QBS scheme, which used Bell states, unitary operations, and so on, in 2015. With the two-photon entangled coding matrix to pass the secret shared key, Lai et al. [30] presented a QBS scheme in 2017. Besides the above QBS schemes with multiple photons, Wang et al. [31] proposed a fair QBS scheme with a single photon in 2010. However, He et al. [32] and Zou et al. [33] found that this scheme was vulnerable to non-forgeability attack. All these QBS schemes [19,20,21,22,23,24,25,26,27,28,29,30,31,32,33] are mainly divided into two broad categories: multi-photon entanglement QBS [19,20,21,22,23,24,25,26,27,28,29,30] and single-photon QBS [31,32,33]. Unlike the proposed QBS schemes with the single photon in [31,32,33], in this paper, we propose a new single-photon QBS scheme encoding with the indistinguishable BB84-state. To guarantee the unconditional security of the proposed scheme, we employ the quantum fingerprint [34] and Zhang et al.’s improved key-controlled-“T” quantum one time pad (QOTP) [35,36] based on Boykin and Roychowdhury’s QOTP [37]. In the proposed scheme, we give the hypothesis that a trusted arbitrator is known by all participants prior to the execution of the protocol. We give a proof of the correctness of the scheme. Security analyses show that the scheme satisfies all the properties of the blind signature: blindness, unforgeability, non-repudiation, unlinkability, and traceability.

The rest of this paper is organized as follows. In Section 2, we introduce some necessary preliminaries. In Section 3, we present the new QBS scheme with the BB84-state. Subsequently, the security analyses of this scheme are presented in Section 4. Finally, some conclusions are drawn in Section 5.

## 2. Preliminary Theory

### 2.1. Properties of the Blind Signature

In general, a blind signature protocol includes four stages, namely message blinding, blind message signing, message unblinding, and signature verification. The original message owner, Alice, first makes a blind transformation on the original message *m* and gets blind message $\tilde{m}$. Alice sends the transformed blind data $\tilde{m}$ to the blind signer, Bob. Then, Bob signs the $\tilde{m}$ and obtains the blind signature $Sign(\tilde{m})$, and the signature is sent back to Alice. Alice strips the blind factor from the $Sign(\tilde{m})$ and gets the signature Sign(m) of the original message *m*. The verifier, Charlie, can verify the correctness of Sign(m).

Generally speaking, a perfect blind signature should satisfy the following properties [18].

- Unforgeability: No one can generate an effective blind signature except the signer himself/herself. This is one of the most basic requirements.
- Non-repudiation: Once a signer has signed a message, he/she cannot deny his/her signature of the message.
- Blindness: Although a signer has signed a message, he/she cannot get the concrete content of the message.
- Unlinkability: Once the signature of the message is public, the signer cannot determine whether he/she has signed the message.
- Traceability: Once a dispute happens, the verifier can trace the signature.

The blind signature satisfying the above properties is considered to be secure. These five properties are the criteria that we should follow in designing blind signatures. The performance of the blind signature is also judged based on these properties.

### 2.2. Quantum Fingerprint

The quantum fingerprint [34], proposed by Buhrman et al. in 2001, is the most appealing protocol in quantum communication complexity (QCC) protocols [38,39]. In this model, two parties (Alice and Bob) select separately inputs *x*, $y\in {\left\{0,1\right\}}^{n}$ and send their quantum fingerprints messages to a third party, called the Referee. The Referee must determine whether *x* equals *y* or not with a small error probability ϵ. To construct a large set of nearly orthogonal quantum states explicitly, consider an error-correcting code $E:{\left\{0,1\right\}}^{n}⟶{\left\{0,1\right\}}^{t}$ where the distance between distinct code words E(x) and E(y) is at least (1−δ)t, here 0<δ<1, c>1, t=cn. For each $x\in {\left\{0,1\right\}}^{n}$, define the $\left(log_{2}\left(t\right)+1\right)$-qubit state:

(1) $$|f\left(x\right)\rangle \overset{def}{=}\frac{1}{\sqrt{t}}\sum_{i=1}^{t}\left|i\rangle \right|E_{i}\left(x\right)\rangle .$$

Note that two distinct code words can be equal in at most δt positions for any x≠y. Consequently, each pair (|f(x)〉,|f(y)〉) has an inner product 〈f(x)|f(y)〉≤δt/t=δ. When the Referee receives the quantum fingerprints |f(x)〉 and |f(y)〉, he measures and outputs the first qubit of the states (H⊗I)(c-SWAP)(H⊗I)|0〉|f(x)〉|f(y)〉. By measuring the first qubit of this state with computational basis {|0〉,|1〉}, the Referee outputs |1〉 (meaning that x=y) with probability $(1-|\langle f\left(x\right)|f\left(y\right)\rangle {|}^{2})/2$. The probability is zero if x=y and at least $(1-\delta^{2})/2$ if x≠y. Thus, the test determines which case holds with one-sided error probability $(1+\delta^{2})/2$. This error probability can be reduced to any ε>0 by setting the fingerprint |f(x)〉 to ${|f\left(x\right)\rangle }^{⊗l}$ for a suitable $l\in O(log_{2}\left(1/\varepsilon \right))$.

So far, several experiments [40,41,42,43,44] have reported successful attempts at implementing the quantum fingerprint.

### 2.3. Improved QOTP Encryption

In 2003, Boykin and Roychowdhury presented the QOTP encryption [37], which is used to encrypt securely *n*-qubit quantum states with the secret classical 2n-bit key. Denote the *n*-qubit quantum message by $|P\rangle =\overset{n}{\underset{i=1}{⨂}}|P_{i}\rangle$ and the *n*-qubit ciphertext message by $|C\rangle =\overset{n}{\underset{i=1}{⨂}}|C_{i}\rangle$, where $|P_{i}\rangle =\alpha_{i}|0\rangle +\beta_{i}|1\rangle$, $|C_{i}\rangle =\alpha_{i}^{\prime }|0\rangle +\beta_{i}^{\prime }|1\rangle$, $|\alpha_{i}{|}^{2}+{\left|\beta_{i}\right|}^{2}$ = $|\alpha_{i}^{\prime }{|}^{2}+{\left|\beta_{i}^{\prime }\right|}^{2}=1$, i∈[1,n]. With the secret classical 2n-bit key *k*, the QOTP encryption $E_{k}$ on |P〉 can be described by $E_{k}|P\rangle$$=\overset{n}{\underset{i=1}{⨂}}\sigma_{x}^{k_{2i}}\sigma_{z}^{k_{2i-1}}|P_{i}\rangle$ and the corresponding decryption $D_{k}$ on |C〉 by $D_{k}|C\rangle$ = $\overset{n}{\underset{i=1}{⨂}}\sigma_{z}^{k_{2i-1}}\sigma_{x}^{k_{2i}}|C_{i}\rangle$. Since the original QOTP [37] is a bitwise protocol, Zhang et al. [35,36] pointed out that it would encounter forgery attack when it is used in the quantum signature. Now, some improved QOTP encryption schemes have been proposed, such as those in [35,36,45]. Since the location permutation of the quantum state is supplemented in the original QOTP, the improved QOTP schemes are no longer the bitwise encryption and provide higher security.

In order to use the improved QOTP to encrypt the classical message, some methods must be used to transform the classical message to the quantum one. Here, we give a simple example to demonstrate this one-to-one correspondence between them. Let us denote the *n*-bit classical message by $M=M_{1}M_{2}\cdots M_{i}\cdots M_{n}$ and its corresponding *n*-qubit quantum one by |M〉 = $\overset{n}{\underset{i=1}{⨂}}{|M_{i}\rangle }_{i}$, where i∈[1,n]. When *i* is odd, encode $M_{i}$ with the rectilinear basis, namely ${|0\rangle }_{i}=|0\rangle$, ${|1\rangle }_{i}=|1\rangle$. When *i* is even, encode $M_{i}$ with the diagonal basis, namely ${|0\rangle }_{i}=|+\rangle$, ${|1\rangle }_{i}=|-\rangle$. According to the parity of the position of each quantum state, a different basis is used to measure the decrypted quantum state, so that the classical message can be recovered when decrypting. Because of the permutation of the quantum position, attacker cannot obtain *M* from the disordered quantum ciphertext by measurement without the secret key. For the sake of brevity and readability, the improved QOTP encryption on classical *M* is denoted by $E_{k}\left(M\right)$ with the secret key *k*. Once the length of the message *M* exceeds *n*, we can divide *M* into several segments of length *n* and then encrypt them separately.

## 3. Quantum Blind Signature Scheme

We first give an encoding method of BB84-state so as to establish the one-to-one correspondence between the classical bit and the quantum one. With the BB84-state encoding, we then propose our new QBS scheme.

### 3.1. BB84-State Encoding

Let $p=\left(p_{1},p_{2},\cdots ,p_{n}\right),q=\left(q_{1},q_{2},\cdots ,q_{n}\right),r=\left(r_{1},r_{2},\cdots ,r_{n}\right)\in {\left\{0,1\right\}}^{n}$, where $p_{i}$ is the *i*th-bit in *p* ($q_{i}$, $r_{i}$ in *q*, *r*, respectively), i∈[1,n]. We give an encoding rule that maps $(p_{i}$, $q_{i})$ to a quantum BB84-state in set {|+〉,|−〉,|0〉,|1〉} and define:

(2) $${|\varphi \rangle }_{p_{i},q_{i}}\overset{def}{=}\left\{\begin{array}{cc} |+\rangle & \left(p_{i}=0,q_{i}=0\right),\\ |-\rangle & \left(p_{i}=1,q_{i}=1\right),\\ |0\rangle & \left(p_{i}=1,q_{i}=0\right),\\ |1\rangle & \left(p_{i}=0,q_{i}=1\right). \end{array}\right.$$

For *n*-qubit ${|\varphi \rangle }_{p,q}$, we define:

(3) $${|\varphi \rangle }_{p,q}\overset{def}{=}{⊗}_{i=1}^{n}{|\varphi \rangle }_{p_{i},q_{i}}.$$

According to Equation (2), it is easy to draw the following conclusions, where ⊕ is the exclusive-or (XOR) operation and the symbol ≅ denotes the equivalence relation between two quantum states, which are different from constant coefficient (for instance, |+〉≅−|+〉).

**Corollary** **1.** ${|\varphi \rangle }_{p_{i},q_{i}}=\left\{\begin{array}{cc} |0\rangle ,|+\rangle & \left(q_{i}=0\right)\\ |1\rangle ,|-\rangle & \left(q_{i}=1\right) \end{array}\right..$

**Corollary** **2.** ${|\varphi \rangle }_{p_{i},q_{i}}=\left\{\begin{array}{cc} |+\rangle ,|-\rangle & \left(p_{i}⊕q_{i}=0\right)\\ |0\rangle ,|1\rangle & \left(p_{i}⊕q_{i}=1\right) \end{array}\right..$

**Corollary** **3.** ${X|\varphi \rangle }_{p_{i},q_{i}}≅\left\{\begin{array}{cc} |+\rangle & \left(p_{i}=0,q_{i}=0\right)\\ |-\rangle & \left(p_{i}=1,q_{i}=1\right)\\ |1\rangle & \left(p_{i}=1,q_{i}=0\right)\\ |0\rangle & \left(p_{i}=0,q_{i}=1\right) \end{array}\right.$.

**Corollary** **4.** ${Z|\varphi \rangle }_{p_{i},q_{i}}≅\left\{\begin{array}{cc} |-\rangle & \left(p_{i}=0,q_{i}=0\right)\\ |+\rangle & \left(p_{i}=1,q_{i}=1\right)\\ |0\rangle & \left(p_{i}=1,q_{i}=0\right)\\ |1\rangle & \left(p_{i}=0,q_{i}=1\right) \end{array}\right.$.

**Corollary** **5.** $H^{r_{i}}{|\varphi \rangle }_{p_{i},q_{i}}={|\varphi \rangle }_{p_{i}⊕r_{i},q_{i}}$.

**Corollary** **6.** $Y^{r_{i}}{|\varphi \rangle }_{p_{i},q_{i}}=\zeta^{r_{i}\left(3+2p_{i}\right)}{|\varphi \rangle }_{p_{i}⊕r_{i},q_{i}⊕r_{i}}≅{|\varphi \rangle }_{p_{i}⊕r_{i},q_{i}⊕r_{i}}$, where ζ is an imaginary unit satisfying $\zeta^{2}=-1$.

### 3.2. The Proposed Quantum Blind Signature Scheme

Substituting the quantum states for all or part of the classical messages, the so-called QBS inherits the definition and signature framework of the classical one. The QBS could achieve unconditional security through a combination of quantum theory and classical cryptography. The proposed signature scheme consists of the initial phase, the blinding phase, the signing phase, the unblinding phase, and the verifying phase.

#### 3.2.1. Initial Phase

According to the different responsibilities in our proposed QBS scheme, there are five different roles: message owner, blind signer, verifier, arbitrator, and external attacker. Let Alice be the original message owner, Bob the blind signer, Charlie the signature verifier, Trent the arbitrator, and Eve the malicious external attacker. In the scheme, we give the hypothesis that Trent is known by all participants prior to the execution of the protocol and acts as the trusted arbitrator. In the remainder of this paper, we abbreviate Alice to A, Bob to B, Charlie to C, Trent to T, and Eve to E just for brevity. C shares each *2n*-bit secret key $k_{AC}$ and $k_{BC}$ with A and B, respectively. T shares each *2n*-bit secret key $k_{AT}$ and $k_{CT}$ with A and C, respectively. At the same time, A shares a *2n*-bit secret key $k_{AB}$ with B. These keys can be generated in a secure manner, e.g., direct face-to-face contact and QKD protocols with unconditional security such as [2,3,4,5].

#### 3.2.2. Blinding Phase

**Step B1.** The message owner A first prepares the original message *m* of the *n*-bit string. Then, A selects randomly the blind factor *w* of the *n*-bit string and blinds *m* to blind message $\tilde{m}$ based on the formula $\tilde{m}=m⊕w$.

**Step B2.** According to Equations (2) and (3), A generates *n*-qubit blind states ${|\varphi \rangle }_{\tilde{m}⊕k_{AB}^{\prime },\tilde{m}}$ with the *n*-bit blind message $\tilde{m}$ and key $k_{AB}^{\prime }$, where the *n*-bit $k_{AB}^{\prime }$ is derived from the *2n*-bit shared key $k_{AB}$ satisfying $k_{{AB}_{i}}^{\prime }=k_{{AB}_{2i}}⊕k_{{AB}_{\left(2i+1\right)}}$, i∈[1,n]. According to Equation (1), A generates quantum fingerprint $|f(\tilde{m})\rangle$ for blind message $\tilde{m}$. With the shared key $k_{AC}$ and $k_{AB}$, A applies the improved QOTP [35,36,45], which is described in Subsection 2.3, to encrypt her classical message *m* and blind factor *w*, and then obtains $E_{k_{AB}}(E_{k_{AC}}\left(m||w)\right)$, where the notation || denotes the concatenation of strings.

**Step B3.** A denotes $Sign_{AB}\overset{def}{=}{\{|\varphi \rangle }_{\tilde{m}⊕k_{AB}^{\prime },\tilde{m}},|f\left(\tilde{m}\right)\rangle ,E_{k_{AB}}(E_{k_{AC}}\left(m||w))\right\}$ and transmits ${Sign_{AB}}^{⊗2}$ to B through the quantum channel, where ${Sign_{AB}}^{⊗2}$ represents two copies of $Sign_{AB}$.

#### 3.2.3. Signing phase

**Step S1.** Analogous to the method in **Step B1**, B obtains the *n*-bit key $k_{AB}^{\prime }$ from the shared *2n*-bit key $k_{AB}$ between A and B. If the $k_{{AB}_{i}}^{\prime }$ is zero, B selects the diagonal basis {|+〉,|−〉}, otherwise rectilinear basis {|0〉,|1〉}. According to this basis rule, B measures all the qubits of the indistinguishable BB84-state ${|\varphi \rangle }_{\tilde{m}⊕k_{AB}^{\prime },\tilde{m}}$ corresponding in $Sign_{AB}$ and gets the blind message ${\tilde{m}}^{\prime }$ with the key $k_{AB}^{\prime }$.

**Step S2.** According to Equation (1), B generates quantum fingerprint $|f({\tilde{m}}^{\prime })\rangle$. B then compares the generated $|f({\tilde{m}}^{\prime })\rangle$ with state $|f(\tilde{m})\rangle$ from $Sign_{AB}$ and judges whether they are equal based on quantum fingerprint theory in [34]. If they are not equal, then B stops the scheme, otherwise draws the conclusion ${\tilde{m}}^{\prime }=\tilde{m}$ and goes on.

**Step S3.** B first selects randomly two *n*-bit strings *u* and *v*. According to Equation (2) and Equation (1), B then generates respectively the QBS BB84-state ${|\varphi \rangle }_{\tilde{m}⊕u,v}$ and quantum fingerprint $|f(u||v||\tilde{m})\rangle$ with *u*, *v*, and $\tilde{m}$. With the shared key $k_{BC}$, B encrypts his strings *u* and *v* and then gets $E_{k_{BC}}\left(u,v\right)$. From the receiving $Sign_{AB}$, B decrypts the $E_{k_{AB}}(E_{k_{AC}}\left(m||w)\right)$ with his shared key $k_{AB}$ and gets $E_{k_{AC}}\left(m||w\right)$, then encrypts it with his shared key $k_{BC}$ and obtains $E_{k_{BC}}(E_{k_{AC}}\left(m||w)\right)$. B denotes $Sign_{BC}\overset{def}{=}\left\{|f(u||v|\right|\tilde{m})\rangle ,E_{k_{BC}}\left(u,v\right),E_{k_{BC}}(E_{k_{AC}}\left(m||w))\right\}$.

**Step S4.** B transmits ${Sign_{BC}}^{⊗2}$ and ${{|\varphi \rangle }_{\tilde{m}⊕u,v}}^{⊗2}$ to A through the quantum channel.

#### 3.2.4. Unblinding Phase

**Step U1.** After receiving the blind signature ${|\varphi \rangle }_{\tilde{m}⊕u,v}$ for blind message $\tilde{m}$ signed by B, A applies $H^{w}$ to ${|\varphi \rangle }_{\tilde{m}⊕u,v}$ with her blind factor *w* and gets $H^{w}{|\varphi \rangle }_{\tilde{m}⊕u,v}$, which is a quantum signature for the original message *m*. With the shared key $k_{AC}$, A generates $E_{k_{AC}}{(H^{w}|\varphi \rangle }_{\tilde{m}⊕u,v})$ and $E_{k_{AC}}\left(m||w\right)$. A denotes $Sign_{AC}\overset{def}{=}\left\{E_{k_{AC}}{(H^{w}|\varphi \rangle }_{\tilde{m}⊕u,v}\right),E_{k_{AC}}\left(m||w)\right\}$.

**Step U2.** A generates $E_{k_{AT}}\left({Sign_{AC}}^{⊗2},{Sign_{BC}}^{⊗2}\right)$ and transmits it to T through the quantum channel.

**Step U3.** T decrypts the received $E_{k_{AT}}\left({Sign_{AC}}^{⊗2},{Sign_{BC}}^{⊗2}\right)$ and gets ${Sign_{AC}}^{⊗2}$ and ${Sign_{BC}}^{⊗2}$. Then, T performs the C-SWAPtest [34] to compare the two copies of $Sign_{AC}$ in ${Sign_{AC}}^{⊗2}$. The same test is also done on ${Sign_{BC}}^{⊗2}$. Once an unequal result of the comparison occurs, T draws the conclusion that the signature is invalid and aborts the process. After T finishes the comparison tests successfully, he preserves one copy of $Sign_{AC}$ and $Sign_{BC}$ to be prepared to solve disputes when they arise in the future. Finally, T generates $E_{k_{CT}}\left(Sign_{AC},Sign_{BC}\right)$ with another copy of $Sign_{AC}$ and $Sign_{BC}$ and transmits it to C through the quantum channel.

#### 3.2.5. Verifying Phase

**Step V1.** C first gets $Sign_{AC}$ and $Sign_{BC}$ from the received $E_{k_{CT}}\left(Sign_{AC},Sign_{BC}\right)$ with his shared key $k_{CT}$. Then, C decrypts the $E_{k_{BC}}(E_{k_{AC}}\left(m||w)\right)$ in $Sign_{BC}$ with his shared key $k_{BC}$, gets $E_{k_{AC}}^{\prime }\left(m||w\right)$, and performs the C-SWAP test [34] to compare it with $E_{k_{AC}}\left(m||w\right)$ in $Sign_{AC}$. If the result of the comparison is not equal, C draws the conclusion that the signature is invalid and aborts the process. Otherwise, C then applies the key-controlled-“T” QOTP to decrypt $E_{k_{AC}}\left(m||w\right)$ with his shared key $k_{AC}$ and finally gets (*m*, *w*).

**Step V2.** After getting $E_{k_{BC}}\left(u,v\right)$ from the $Sign_{BC}$, C decrypts $E_{k_{BC}}\left(u,v\right)$ with his shared key $k_{BC}$ and then gets (*u*, *v*).

**Step V3.** From the received $Sign_{AC}$, C decrypts $E_{k_{AC}}{(H^{w}|\varphi \rangle }_{\tilde{m}⊕u,v})$ with his shared key $k_{AC}$ and gets $H^{w}{|\varphi \rangle }_{\tilde{m}⊕u,v}$. With the *m* obtained in ***Step V1***, C applies $H^{m}$ to $H^{w}{|\varphi \rangle }_{\tilde{m}⊕u,v}$ and gets $H^{m}H^{w}{|\varphi \rangle }_{\tilde{m}⊕u,v}$.

**Step V4.** With the *u*, *v* obtained in ***Step V2***, C performs single-particle measurements on the *n*-qubit $H^{m}H^{w}{|\varphi \rangle }_{\tilde{m}⊕u,v}$ obtained in ***Step V3*** and gets $u^{\prime },v^{\prime }$. The rules of measurement are as follows. According to Corollary 2, C uses diagonal basis {|+〉,|−〉} to measure the BB84-state if $u_{i}⊕v_{i}=0$, otherwise rectilinear basis {|0〉,|1〉}. Based on the measurement result and Equation (2), C can deduce the corresponding $u_{i}^{\prime }$, $v_{i}^{\prime }$. C aborts the process if $u_{i}\neq u_{i}^{\prime }$ or $v_{i}\neq v_{i}^{\prime }$ for some i∈[1,n], otherwise goes on.

**Step V5.** C generates quantum fingerprint |f(u||v||(m⊕w))〉 with the deduced (*u*, *v*) and (*m*, *w*) and then compares it with $|f(u||v||\tilde{m})\rangle$ in $Sign_{BC}$ from B. If the result of comparison is equal, C draws the conclusion that the signature is valid, otherwise declares that the signature is not valid.

**Step V6.** According to Equation (2), C regenerates the quantum BB84-state signature ${|\varphi \rangle }_{m⊕u,v}$ with the known *m*, *u*, and *v*. C announces publicly the QBS correctness and declares the signature ${\{m,|\varphi \rangle }_{m⊕u,v}\}$ to the public.

**Figure 1:**
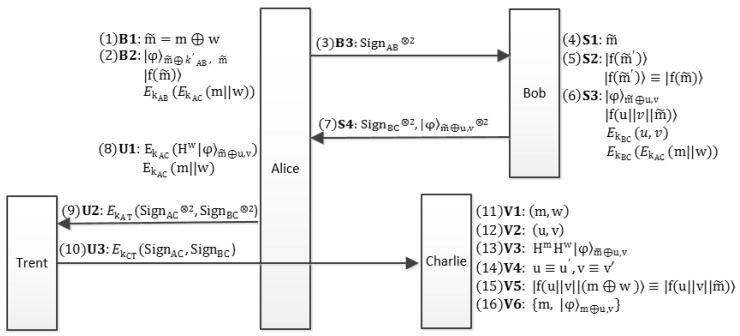
The flow-process diagram of the proposed QBS scheme. Alice blinds the message *m* with blind factor *w* and passes $\tilde{m}$ to Bob. With the proposed encoding method of the BB84-state, Bob signs $\tilde{m}$ with *u* and *v* and then sends the blind signature ${|\varphi \rangle }_{\tilde{m}⊕u,v}$ back to Alice. Two copies of the unblinding signature are sent to Trent. After Trent’s identical verification for the two signatures, one of the signatures is transmitted to Charlie. Once Charlie verifies the validity of the signature, he publishes the signature ${\{m,|\varphi \rangle }_{m⊕u,v}\}$.

The whole flow-process diagram of the proposed QBS scheme is given in Figure 1.

## 4. Security Analyses

In this section, we show that the proposed scheme is correct and satisfies the properties of blind signatures described in the preliminary section.

### 4.1. Correctness

**Theorem** **1.** The QBS scheme is correct.

**Proof.**  We prove the correctness of the scheme in two cases.

(1) B can correctly recover the blind message $\tilde{m}$ from A.

In the blinding phase, B received the ${|\varphi \rangle }_{\tilde{m}⊕k_{AB},\tilde{m}}$ from A. According to Corollary 2, the correct chosen basis (diagonal or rectilinear) to measure ${|\varphi \rangle }_{{\tilde{m}}_{i}⊕k_{{AB}_{i}},{\tilde{m}}_{i}}$ is determined by $\left({\tilde{m}}_{i}⊕k_{{AB}_{i}}\right)⊕{\tilde{m}}_{i}=k_{{AB}_{i}}$. In the cases in which A is an honest blind message sender and no eavesdropper E exists in the quantum channel, as long as B measures ${|\varphi \rangle }_{\tilde{m}⊕k_{AB},\tilde{m}}$ in the correct basis determined by the shared key $k_{AB}$, B will always get the correct blind message $\tilde{m}$ with the probability one by comparison of his measurement results with Equation (2). However, if A is not an honest blind message sender or eavesdropper E exists in the quantum channel, B will find, with high probability, a contradiction with the measurement results and aborts.

(2) C can correctly validate the quantum signature $H^{w}{|\varphi \rangle }_{\tilde{m}⊕u,v}$ for A’s original message *m*.

After recovering the blind message $\tilde{m}$ from ${|\varphi \rangle }_{\tilde{m}⊕k_{AB},\tilde{m}}$, B signs $\tilde{m}$ with his *u* and *v* and gets QBS ${|\varphi \rangle }_{\tilde{m}⊕u,v}$ based on Equation (2). Once A gets the QBS ${|\varphi \rangle }_{\tilde{m}⊕u,v}$ from B, she strips the blind factor *w* by applying $H^{w}$ to ${|\varphi \rangle }_{\tilde{m}⊕u,v}$ and gets quantum signature $H^{w}{|\varphi \rangle }_{\tilde{m}⊕u,v}$. In fact, according to Corollary 5, A obtains the result:

$$\begin{array}{c} H^{w}{|\varphi \rangle }_{\tilde{m}⊕u,v}={|\varphi \rangle }_{(\tilde{m}⊕u)⊕w,v}={|\varphi \rangle }_{(m⊕w)⊕u⊕w,v}={|\varphi \rangle }_{m⊕u,v}. \end{array}$$

In ***Step V3***, after receiving $E_{k_{AC}}{(H^{w}|\varphi \rangle }_{\tilde{m}⊕u,v})$ from A, C decrypts it and gets $H^{w}{|\varphi \rangle }_{\tilde{m}⊕u,v}$ with his shared key $k_{AC}$, then applies $H^{m}$ to $H^{w}{|\varphi \rangle }_{\tilde{m}⊕u,v}$ to generate $H^{m}H^{w}{|\varphi \rangle }_{\tilde{m}⊕u,v}$, i.e.,

$$\begin{array}{c} H^{m}H^{w}{|\varphi \rangle }_{\tilde{m}⊕u,v}=H^{m}{|\varphi \rangle }_{m⊕u,v}={|\varphi \rangle }_{(m⊕u)⊕m,v}={|\varphi \rangle }_{u,v}. \end{array}$$

With the decrypted *u* and *v* from B, C selects a suitable basis and measures $H^{m}H^{w}{|\varphi \rangle }_{\tilde{m}⊕u,v}$(namely, ${|\varphi \rangle }_{u,v}$). It is obvious that the measurement results must match *u* and *v*. Thus, we can draw the conclusion that C can correctly validate the quantum signature $H^{w}{|\varphi \rangle }_{\tilde{m}⊕u,v}$ for A’s original message *m*. □

### 4.2. Against External Attack

It is impossible for external attacker E to attack a legitimate signature. Being external, the attacker has less available resources than A or B. The only way for him/her to obtain information is to intercept the quantum states or eavesdrop on the quantum channel. In the proposed scheme, there are three forms of quantum states on the quantum channel: quantum fingerprint states, BB84-states, and encrypted quantum states.

For the quantum fingerprint in the quantum channel, it is impossible for E to deduce conversely the original input on the basis of [34]. At the same time, if any quantum states are measured or replaced, this attack is detected by participants’ comparison of quantum fingerprint states. Therefore, it is impossible for E to forge the scheme by attacking the quantum fingerprint states.

Both BB84-states and encrypted quantum states, which are *n*-qubit tensor products, consist of elements in set {|+〉,|−〉,|0〉,|1〉}. Assuming that the secret keys and signature parameters are uniformly distributed, each qubit is randomly located in one of two conjugate bases. Thus, the quantum states are essentially the same as the BB84 QKD one. According to the quantum indistinguishability, non-cloning, and immeasurability, E cannot distinguish the nonorthogonal states. E cannot perform the correct unitary operation for each photon. In terms of mathematical probability, he/she only speculates each photon state with the correct probability $\frac{1}{4}$. Therefore, the probability of misjudgment for n photons is:

(4) $$P=1-{\left(\frac{1}{4}\right)}^{n}.$$

Obviously, this probability infinitely tends to one with the increase of *n*. E cannot obtain any message from the transmitted particles yet. Consider the density matrix of *n* particles,

(5) $$\rho ={\left(\frac{1}{4}\right)}^{n}(|+\rangle \langle +|+|-\rangle \langle -|+|0\rangle \langle 0|+|1\rangle {\langle 1|)}^{⊗n}=\frac{1}{2^{n}}I.$$

This illustrates that the quantum states distribute in a uniform way so that no information might be leaked to the eavesdropper E. Consequently, external attack would not take effect.

### 4.3. Blindness

In the blinding phase, A sends the BB84-state ${|\varphi \rangle }_{\tilde{m}⊕k_{AB},\tilde{m}}$, which contains blind message $\tilde{m}$, to B. To measure ${|\varphi \rangle }_{\tilde{m}⊕k_{AB},\tilde{m}}$ using the corresponding basis matching the shared key $k_{AB}$, B can recover the blind message $\tilde{m}$ from A. For $\tilde{m}=m⊕w$; thus, B cannot recover *m* directly from known $\tilde{m}$ without *w*.

However, the blind signer B has two strategies to find some original message if A’s quantum signature is transmitted in the form $H^{w}{|\varphi \rangle }_{\tilde{m}⊕u,v}$ (namely,${|\varphi \rangle }_{m⊕u,v}$) in $Sign_{AC}$. B’s first strategy is to measure A’s ${|\varphi \rangle }_{m_{i}⊕u_{i},v_{i}}$ with computational basis {|0〉,|1〉} or diagonal basis {|+〉,|−〉}. Suppose $u_{i}=v_{i}=0$; once B measures the ${|\varphi \rangle }_{m_{i}⊕u_{i},v_{i}}$ (namely, ${|\varphi \rangle }_{m_{i},0}$) with the computational basis and gets the measurement result |1〉, he can come to the conclusion that ${|\varphi \rangle }_{m_{i},0}$ cannot be |0〉 and must be |+〉. This shows that Alice’s original message must be $m_{i}=0$. On average, Bob’s strategy thus reveals $\frac{1}{4}n$ bits of A’s original message. The result is the same for the use of the diagonal basis. B’s second strategy is to perform the C-SWAP test [34] between his ${|\varphi \rangle }_{{\tilde{m}}_{i}⊕u_{i},v_{i}}$ and A’s ${|\varphi \rangle }_{m_{i}⊕u_{i},v_{i}}$ if B can certainly confirm that A’s stripped signature ${|\varphi \rangle }_{m_{i}⊕u_{i},v_{i}}$ corresponds to his blind signature ${|\varphi \rangle }_{\tilde{m_{i}}⊕u_{i},v_{i}}$. According to the comparison result, B can come to the conclusion that $m_{i}={\tilde{m}}_{i}$ ($m_{i}={\tilde{m}}_{i}⊕1$) if ${|\varphi \rangle }_{{\tilde{m}}_{i}⊕u_{i},v_{i}}$ = ${|\varphi \rangle }_{m_{i}⊕u_{i},v_{i}}$ (${|\varphi \rangle }_{{\tilde{m}}_{i}⊕u_{i},v_{i}}$≠${|\varphi \rangle }_{m_{i}⊕u_{i},v_{i}}$), and then, B can get A’s original message $m_{i}$.

To avoid the two extreme strategies of B existing in his blind signature and A’s stripped one, the quantum signature $H^{w}{|\varphi \rangle }_{\tilde{m}⊕u,v}$ is encrypted with the key $k_{AC}$ in the proposed scheme, and then, $E_{k_{AC}}{(H^{w}|\varphi \rangle }_{\tilde{m}⊕u,v})$ is transmitted to C in **Step U2**. In such circumstances, the two strategies of B become invalid. Thus, our proposed scheme meets the standard of blindness.

### 4.4. Unforgeability

There are two kinds of forgeries. One forgery is done by the internal participants and the other by the external attacker E. The attacks of the internal participants involve message owner A, blind signer B, and the signature verifier C. With the following analyses, it can be shown that the two kinds of forgery cannot forge legitimate signatures so as to achieve the purpose of passing C’s verification.

The message owner A cannot forge the quantum signature. For the blind message $\tilde{m}=m_{1}⊕w_{1}$ corresponding to original message $m_{1}$ and blind factor $w_{1}$, A would reach her purpose of forgery if she succeeds in forging message pair ($m_{2}$, $w_{2}$) to replace the true message ($m_{1}$, $w_{1}$) and making C validate it. Obviously, $m_{1}⊕w_{1}$ must be equal to $m_{2}⊕w_{2}$, otherwise C will find the inconformity in **Step V5**. There are two ways for A to forge. One way is that A prepares the original message pair ($m_{1}$, $w_{1}$) in **Step B1** and $E_{k_{AB}}\left(E_{k_{AC}}\left(m_{1},w_{1}\right)\right)$ in **Step B2** and at the same time generates $E_{k_{AC}}{(H^{w_{2}}|\varphi \rangle }_{\tilde{m}⊕u,v})$ and $E_{k_{AC}}\left(m_{2},w_{2}\right)\}$ in **Step U1**. In this way, C will find that ($m_{1}$, $w_{1}$) is not equal ($m_{2}$, $w_{2}$) and abort this signature. Thus, this strategy is unsuccessful. Another way for A’s forgery is to generate $E_{k_{AC}}{(H^{w_{2}}|\varphi \rangle }_{\tilde{m}⊕u,v})$ and $E_{k_{AC}}\left(m_{1},w_{1}\right)\}$ in **Step U1**. This way can pass C’s examination in **Step V1**, but C would still find this strategy in **Step V3** and **Step V4**. In **Step V3**, C applies $H^{m_{1}}$ to $H^{w_{2}}{|\varphi \rangle }_{\tilde{m}⊕u,v}$ and gets:

$$\begin{array}{c} H^{m_{1}}H^{w_{2}}{|\varphi \rangle }_{\tilde{m}⊕u,v}={|\varphi \rangle }_{m_{1}⊕w_{2}⊕\tilde{m}⊕u,v}={|\varphi \rangle }_{m_{1}⊕w_{2}⊕\left(m_{1}⊕w_{1}\right)⊕u,v}={|\varphi \rangle }_{w_{1}⊕w_{2}⊕u,v}. \end{array}$$

Obviously, if the $w_{1}$ is not equal $w_{2}$ and thus $w_{1}⊕w_{2}⊕u$ is not equal to *u*, C would find this strategy with the examination in **Step V4** for this way. Thus, the two forgery ways for A are not effective, and the unforgeability of the proposed scheme holds.

It is impossible for the blind signer B to forge a legitimate signature. The forgery way of B is to masquerade as the message owner A to sign the message alone and attempt to let the verifier C verify this forged signature. At first, B generates the forged message $m^{\prime }$ and the blind parameter $w^{\prime }$. Then, B takes the place of A to get the unblinding quantum signature $H^{w^{\prime }}{|\varphi \rangle }_{\tilde{m^{\prime }}⊕u,v}={|\varphi \rangle }_{m^{\prime }⊕u,v}$ in **Step U1**. According to our scheme, $m^{\prime }$ and $w^{\prime }$ must be encrypted with the shared key $k_{A^{\prime }C}$, and then, $E_{k_{A^{\prime }C}}\left(m^{\prime },w^{\prime }\right)$ will be transmitted to C. In **Step V1**, C decrypts $E_{k_{A^{\prime }C}}\left(m^{\prime },w^{\prime }\right)$ with the shared key $k_{AC}$, but C cannot get the correct $m^{\prime }$ and $w^{\prime }$ because of B’s random guess key $k_{A^{\prime }C}$. In **Step V4**, C will find the forgery trick because the equations are not satisfied.

The signature verifier C cannot forge the quantum signature. After the arbitrator T receives the two copies of signatures $E_{k_{AT}}\left({Sign_{AC}}^{⊗2},{Sign_{BC}}^{⊗2}\right)$ encrypted with the shared key $k_{AT}$ from A, he retains one copy and then encrypts another to C with shared key $k_{CT}$. If C forges a blind signature and tries to cheat the message owner A, it will cause a dispute. In this dispute, T can judge that C is the forger. This is because T retains a legitimate signature. According to the signature data provided by C, T can regenerate C’s signature. With the C-SWAP test [34], T can compare the preserved signatures with C’s forged signature and will find the inconformity, so C’s forgery strategy fails. Therefore, with the help of the trusted arbitrator T, C’s forgery strategy is not feasible.

### 4.5. Non-Repudiation

There are three possible ways for participants to repudiate the quantum signature afterwards. The first way is that A, the original message owner, repudiates that she has ever blinded message *m* to $\tilde{m}$ with random *w* in the blinding phase and stripped blind factor *w* from blind signature in the unblinding phase. The second way is that B, the blind signer, repudiates that he has signed the blind message $\tilde{m}$ with the random parameters *u* and *v*. The third way is that the signature verifier C denies that he has verified the legitimate signature from message owner A.

For A, she cannot repudiate her blinding behavior because she transmits the blind message $\tilde{m}$ encoding the quantum BB84-state with the shared key $k_{AB}$ and the quantum fingerprint $|f(\tilde{m})\rangle$ to B in the blinding phase. She cannot also repudiate her unblinding behavior because she encrypts the blind factor *w* with shared key $k_{AC}$ and transmits $E_{k_{AC}}\left(m||w\right)$ to C in the unblinding phase.

For B, he encrypts his blind signature parameters *u* and *v* with the shared key $E_{k_{BC}}$ and passes $E_{k_{BC}}\left(u,v\right)$ to C, so he cannot repudiate the behavior of his choosing of the parameters. Meanwhile, in the verifying phase, C generates |f(u||v||(m⊕w))〉 and compares it to the receiving $\left|f(u||v||\tilde{m}\right)$ from B and thus further validates the signature parameters {w,u,v} and denies A’s repudiation and B’s one as a whole.

For C, his undeniable attribute is determined by his declaration behavior in the verifying phase. That is to say, in several consecutive judgments, once C announces the correct judgment of the signature, he cannot deny all previous declarations including this one. The received $E_{k_{CT}}\left(Sign_{AC},Sign_{BC}\right)$, which is encrypted with his shared key $k_{CT}$, comes from the trusted arbitrator T. In **Step V1** of the verifying phase, it indicates that the $E_{k_{AC}}\left(m||w\right)$ is equal in $Sign_{AC}$ and $Sign_{BC}$. C confirms the fact of C not aborting the signature verification procedure. If so, C can not deny this verification step. In **Step V4** of the verifying phase, C performs single-particle measurements on the quantum signature and deduces B’s signature parameters $u^{\prime }$ and $v^{\prime }$. C would aborts the process if he finds disagreement between the derivative results $\left(u^{\prime },v^{\prime }\right)$ and received results (u,v). Thus, C cannot deny his actions in this step. In **Step V5** of the verifying phase, C validates the quantum fingerprint and judge A’s blind parameter *w*. Similarly, his announcement for A’s blindness cannot be disavowed. In the whole process, it shows that C has accepted the process of signature verification and cannot deny his validation fact if C does not abandon the verification in the verifying phase.

### 4.6. Unlinkability

Once the original message *m* and its quantum signature ${|\varphi \rangle }_{m⊕u,v}$ are public, the proposed scheme guarantees that the blind signer B cannot determine whether the open message *m* is associated with the blind message $\tilde{m}$ or not. Suppose C opens publicly two different signatures ${\{m_{1},|\varphi \rangle }_{m_{1}⊕u,v}\}$ and ${\{m_{2},|\varphi \rangle }_{m_{2}⊕u,v}\}$; the blind signer B can certainly validate the correctness of each signature with the open $m_{1}$ ($m_{2}$) and his secret *u* and *v*. Meanwhile, B can recover his blind signature ${\{\tilde{m},|\varphi \rangle }_{\tilde{m}⊕u,v}\}$ with A’s blind message $\tilde{m}$ and B’s (u,v). Because B does not know A’s blind factors $w_{1}$ and $w_{2}$, facing the open messages $m_{1}$ and $m_{2}$, B cannot be sure whether his blind message $\tilde{m}$ is associated with the open message $m_{1}$ or $m_{2}$ besides only verifying the correctness of these signatures. Thus, this proposed scheme is shown to admit the property of unlinkability.

### 4.7. Traceability

The scheme is verified to satisfy the security demand of full traceability. Under the supervision of trusted arbitrator T, the verifier C is provided with traceability when the dispute occurs. Because the message owner A’s blind parameter *w* and original message *m* are all encrypted and transmitted to C, C can trace the whole original message sender A’s process. At the same time, C is traceable to B’s blind signature process because B’s blind signature parameters *u* and *v* are also encrypted and transmitted to C. Thus, this signature scheme satisfies the condition of traceability.

## 5. Conclusions

In this paper, we presented a new provable QBS scheme with the nonorthogonal single-photon BB84-state. We supposed that the arbitrator was trusted by everyone. Following the classical blind signature, our scheme consisted of the initial, blinding, signing, unblinding, and verifying phases. The original message owner was responsible for the blinding and unblinding messages. The duty of blind signer was to sign the blinding message without knowing the original message. When a dispute occurred, the trusted arbitrator could open the quantum signature to identify the original message owner, blind signer, or signature message verifier. Based on quantum indistinguishability, the quantum encryption algorithm, the quantum fingerprint, and so on, the scheme provided unconditional security. Differing from the previous QBS schemes with some security vulnerability in basis security requirements, the security analyses showed that the proposed scheme satisfied the five properties of the blind signature protocol. Therefore, our scheme could be safely applied in some special environments.

Based on the current development level of quantum experiment technology, it is not ideal enough to achieve multi-photon quantum entanglement in practice. Our scheme uses only the single-photon BB84-state instead of quantum multi-photon entanglement states. Therefore, under the current technology and experiment condition, our scheme is realizable. The technical threshold is low, so our scheme is practical and feasible.

Until now, all the current quantum signature schemes, including our proposed QBS scheme, have used quantum symmetric encryption technology, which would lead to some problems, such as the management, storage, and transmission of shared keys. It will be more convenient to realize the quantum signature if it does not rely on the quantum encryption technology, but quantum public-key cryptography as the classical signature. However, the current quantum public-key cryptography is still in the initial stage of research and not yet mature for use in the quantum digital signature. It is believed that the quantum signature will become more concise and easier to realize with the continuing development of quantum public-key cryptography in the future.
